# Neurocognitive and neurophysiological consequences of sleep-disordered breathing in bronchiectasis: the role of respiratory rehabilitation

**DOI:** 10.3389/fresc.2026.1846233

**Published:** 2026-07-22

**Authors:** Elvia Giovanna Battaglia, Gloria Leonardi, Paolo Innocente Banfi, Eleonora Volpato

**Affiliations:** 1 Sleep Center, Cardio-Pulmonary Rehabilitation, IRCCS Don Carlo Gnocchi Foundation, Milan, Italy; 2 Cardio-Pulmonary Rehabilitation, IRCCS Don Carlo Gnocchi Foundation, Milan, Italy; 3 Dipartimento di Psicologia, Università Cattolica del Sacro Cuore, Milan, Italy

**Keywords:** bronchiectasis, chronic respiratory disease, cognitive function, multidisciplinary care, non-invasive ventilation, obstructive sleep apnea, positive airway pressure, pulmonary rehabilitation

## Abstract

Bronchiectasis (BE) is a chronic respiratory disease characterized by a vicious cycle of irreversible bronchial dilatation and persistent respiratory infections which results in progressive functional impairment and reduced quality of life. Increasing attention has been directed toward comorbidities that may aggravate disease burden, including sleep-disordered breathing (SDB), which remains underrecognized in this population. Emerging evidence suggests that SDB, particularly obstructive sleep apnea (OSA), is highly prevalent in BE and may contribute to adverse outcomes through mechanisms such as intermittent hypoxia, systemic inflammation, microbiome alterations, and ventilatory instability. This narrative review synthesizes current evidence on the relationship between BE and SDB, with a focus on the underlying pathophysiological mechanisms, the neurophysiological and cognitive consequences and the implications for rehabilitation. A comprehensive literature search was conducted using PubMed, Embase, and the Cochrane Library, supplemented by evidence from related chronic respiratory diseases. Available data indicate that sleep disturbances are common in BE and are associated with impaired daytime functioning, reduced quality of life as well as increased symptom burden, independent of disease severity. Rehabilitation interventions, in particular pulmonary rehabilitation, positive airway pressure (PAP), and non-invasive ventilation (NIV), may offer clinically meaningful benefits by enhancing gas exchange, improving sleep quality and patient-reported outcomes. However, high-quality evidence specific to BE populations remains limited. In conclusion, we consider that recognizing SDB as a potentially modifiable trait in bronchiectasis highlights the need for integrated, multidisciplinary management strategies. Future research should prioritize prospective studies to clarify the role of targeted rehabilitation interventions and to support evidence-based clinical practice.

## 1 Introduction

Bronchiectasis (BE) is a chronic respiratory condition characterized by destruction of the elastic and muscular components of the bronchial walls, leading to irreversible bronchial dilatation. These structural abnormalities promote mucus retention and create a favourable environment for secretion stagnation, creating a self-perpetuating vicious cycle of chronic infection and airway inflammation that drives disease progression and leads to recurrent infections and persistent respiratory symptoms (1, 2).

Although still underdiagnosed, BE has gained increasing attention in recent years due to its rising incidence and prevalence—particularly among the elderly—as well as its significant healthcare burden (3–5). Economic burden and clinical impact are especially high in patients with frequent exacerbations and *Pseudonomas aeruginosa* colonisation (6, 7).

BE is increasingly recognized as part of the broader spectrum of chronic airway diseases (8), frequently coexisting with conditions such as Chronic Obstructive Pulmonary Disease (COPD) (9). Several high-resolution computed tomography studies have demonstrated a substantial prevalence of BE among patients with COPD, and the coexistence of these conditions defines a distinct clinical phenotype characterized by greater symptom burden, increased functional limitation, more frequent exacerbations, and poorer prognosis compared with either disease alone (10). Importantly, sleep disturbances are also highly prevalent in BE, with approximately 50%–60% of people who are clinically stable reporting poor sleep quality and 40%–60% presenting with coexisting Obstructive Sleep Apnea (OSA) (11). OSA is increasingly recognized as a systemic inflammatory condition. Through intermittent hypoxia, immune dysregulation, and alterations in the airway microbiome, OSA may increase susceptibility to respiratory infections and contribute to disease progression in chronic respiratory disorders, including BE (12). These findings suggest that sleep-related disorders may represent an underrecognized but potentially modifiable comorbidity, highlighting the importance of identifying treatable traits to optimize disease management, clinical outcomes, and quality of life.

Given the frequent coexistence of BE with other respiratory diseases, increasing attention has been directed toward the potential contribution of Sleep-Disordered Breathing (SDB) to disease burden and clinical outcomes. This interest is supported by evidence from other chronic airway diseases, particularly the well-established COPD–OSA overlap syndrome (13). SDB encompasses a spectrum of abnormal respiratory events occurring during sleep, with OSA representing its most common form. Importantly, multiple mechanisms associated with BE—including airway obstruction, mucus retention, chronic inflammation, and impaired gas exchange—may predispose patients to sleep-related respiratory disturbances.

Beyond respiratory impairment, sleep disruption and intermittent hypoxia have been increasingly recognized as contributors to neurophysiological and cognitive dysfunction through mechanisms involving systemic inflammation, oxidative stress, autonomic dysregulation, and altered sleep architecture (14). In chronic respiratory diseases, these alterations may negatively affect daytime functioning, symptom perception, quality of life, and disease self-management (14). In this context, OSA in patients with BE may present distinct features compared with the general population. The aim of this narrative review is to explore the potential relationship between BE, SDB, and their potential neurophysiological and cognitive consequences, highlighting their possible clinical implications for symptom burden, quality of life, and disease management.

## 2 Materials and methods

A comprehensive literature search was performed using PubMed, Embase, and the Cochrane Library. Articles addressing bronchiectasis, sleep-related respiratory disorders, nocturnal respiratory impairment, pulmonary rehabilitation, cognitive dysfunction, and associated clinical outcomes were considered. Given the limited number of studies specifically investigating the relationship between bronchiectasis, sleep disturbances, and neurocognitive outcomes, relevant evidence from other chronic respiratory diseases, including chronic obstructive pulmonary disease and cystic fibrosis, was also reviewed when appropriate. The selected literature was critically evaluated and integrated to provide a narrative overview of current evidence, highlight potential pathophysiological mechanisms, and identify gaps and future research priorities in this field.

### 2.1 Bronchiectasis and sleep apnea

No clear relationship between BE and SBD have been established. However, it has been recently shown that approximately half of all patients with BE demonstrate PSG-confirmed OSA (11). People with BE exhibit multiple pathophysiological features predisposing to SDB, including chronic airway inflammation, ventilatory impairment (both obstructive and restrictive), and mucus retention due to defective mucociliary clearance and excessive mucus plugging (15–17).

Despite extensive investigation in obstructive airway diseases such as COPD and asthma, SDB remains underexplored in BE. Available data suggest that 40%–60% of people with BE may have coexisting OSA, with higher apnea–hypopnea index (AHI) values observed in those colonized by *Pseudomonas. aeruginosa* (18).

Evidence from polysomnographic studies remains limited. Two cohorts from Brazil and Turkey (*n* = 49 and *n* = 43) reported OSA prevalence between 41% and 56%, predominantly mild in severity. Older age, male sex, increased neck circumference, and *Pseudomonas aeruginosa* colonization emerged as risk factors, whereas body mass index, lung function, and exacerbation history were not significantly associated (18, 19). Disease severity also appears relevant: ≥3 annual exacerbations were associated with longer snoring duration, lower SpO₂ nadir, and increased nocturnal hypoxemia (19).

Furthermore, sleep disturbances in people with BE are often associated with impaired daytime functioning and reduced quality of life. Previous studies reported poor sleep quality in up to 57% of patients, with nocturnal cough, sputum production, and depressive symptoms emerging as major contributing factors, independently of lung function impairment or disease severity indices (19–21).

Polysomnographic studies further demonstrated a high prevalence of OSA, nocturnal hypoxemia, and sleep fragmentation in this population, with excessive daytime sleepiness frequently reported despite predominantly mild OSA severity. Interestingly, patients with coexisting OSA showed higher rates of Pseudonomas aeruginosa colonization, suggesting a possible interaction between nocturnal respiratory dysfunction, inflammation, and disease burden (19).

Altogether, these findings support the concept that sleep disruption in BE is multifactorial and may involve respiratory, psychological, and neurophysiological mechanisms. However, the limited availability of objective sleep assessments still hampers a clear characterization of SDB phenotypes and their clinical implications in this population.

The coexistence of BE with other chronic respiratory diseases further amplifies the burden of SDB. In COPD populations, BE is more prevalent in patients with COPD–OSA overlap syndrome than in those without OSA (43% vs. 19%) and is associated with more severe nocturnal hypoxemia and systemic inflammation (9).

Insights also emerge from primary ciliary dyskinesia (PCD), a condition frequently associated with BE. Studies in pediatric populations demonstrate higher rates of SDB, worse sleep quality, and significant nocturnal respiratory abnormalities—including increased apnoea–hypopnoea indices and oxygen desaturation—compared with healthy controls (22, 23). Notably, subjective sleep questionnaires did not correlate with polysomnographic findings, and bronchiectasis severity was inversely associated with nocturnal oxygen saturation (22, 23).

Overall, current evidence suggests that SDB is common yet under-recognized in BE, with atypical clinical presentation and significant clinical implications. These findings highlight the need for increased awareness, systematic screening, and further studies using objective diagnostic tools to better define the prevalence, mechanisms, and clinical impact of SDB in this population.

### 2.2 Etiopathogenic mechanisms

The coexistence of BE and SDB is likely multifactorial, involving structural airway abnormalities, chronic infection, inflammation and altered ventilatory control. These mechanisms interact synergistically, promoting disease progression and increasing clinical burden.

#### 2.2.1 Inflammation, infection and microbiome

Upper airway inflammation represents a pivotal pathophysiological link between BE and SDB. Histopathological studies in patients with OSA have described the presence of subepithelial edema and inflammatory cell infiltration in the upper airway; these changes resemble those observed in chronic respiratory infections and contribute to airway narrowing and increased collapsibility (24). Although the initiating factors are different, chronic neutrophilic airway inflammation in BE may be responsible for a similar pro-inflammatory environment, suggesting that both OSA and BE converge on shared mechanisms that predispose to upper airway dysfunction and sleep-related respiratory events.

Inflammation, chronic infection, hypoxia, and microbial dysbiosis appear to reinforce one another in a self-perpetuating cycle. In BE, persistent bacterial colonization and chronic airway inflammation increase metabolic oxygen demand while impaired ventilation limits oxygen delivery to diseased lung regions, promoting both intermittent and sustained hypoxia (25). Hypoxia, in turn, leads to oxidative stress, which causes structural airway damage, impaired epithelial integrity, and further amplification of inflammatory pathways (25). When SDB coexists with BE, recurrent episodes of intermittent hypoxia may further intensify this inflammatory response, whereas OSA-related immune dysfunction has been associated with impaired host defense and increased susceptibility to infection-related exacerbations (26).

The airway microbiome is increasingly recognized as an important element of these processes. Dysbiosis promotes both local and systemic inflammation, alters immune homeostasis and may contribute to disease progression. In OSA, alterations of both the gut and airway microbiota have consistently been associated with enhanced inflammatory activation as well as disease severity (27, 28). Healthy individuals typically exhibit nasal microbial communities dominated by *Staphylococcus, Propionibacterium,* and *Corynebacterium*, whereas patients with OSA demonstrate increased abundance of *Streptococcus, Prevotella,* and *Veillonella*, accompanied by higher circulating inflammatory markers (29). Similarly, studies of the lower airway microbiome in OSA have reported enrichment of *Proteobacteria* and *Fusobacteria* with depletion of *Firmicute.* These chances are accompanied by increased interleukin-6 (IL-6) and interleukin-8 (IL-8) serum levels and neutrophilic inflammation (28, 30–32). Evidence on microbiome alterations specific to subjects affected by BE remains limited and reflects the underlying chronic infective nature of the disease rather than sleep-related mechanisms. Nevertheless, available studies suggest that persistent airway dysbiosis contributes to chronic neutrophilic inflammation, recurrent infections and disease progression, indicating substantial overlap with the inflammatory pathways described in OSA (29, 32). Although the microbial composition differs between the two diseases, both are characterized by disruption of host–microbiome interactions, sustained immune activation and impaired mucosal defence, supporting the hypothesis that dysbiosis represents a shared mechanistic pathway linking chronic airway inflammation and SDB.

Consistent with this integrated model, chronic infection-related inflammation and airway edema further induces upper airway collapsibility, thereby facilitating the development and persistence of SDB (33). Although clinical evidence remains limited, an increased prevalence of OSA has been reported in patients with non-cystic fibrosis bronchiectasis (NCFB). In these patients, impaired mucociliary clearance, bronchial dilatation, chronic bacterial colonization, and persistent airway inflammation may collectively increase airflow obstruction and susceptibility to SDB (32). Conversely, intermittent hypoxia associated with SDB promotes systemic inflammation through increased serum levels of TNF-α and IL-6 production (34–36), potentially contributing to lung injury, recurrent infective exacerbations, and progression of chronic respiratory disease (36). Together, these observations support a bidirectional interaction in which BE promotes conditions favouring SDB, while SDB may enhance the inflammatory and infective processes underlying BE progression.

#### 2.2.2 Ventilatory instability

Ventilatory instability represents another key mechanism underlying the bidirectional relationship between BE and SDB. Sleep fragmentation and recurrent hypoxic events disrupt normal respiratory control and promote unstable breathing patterns characterized by repetitive arousals, which amplify post-event ventilatory responses and perpetuate respiratory instability (29, 37, 38).

A major determinant of this instability is loop gain, a measure of the sensitivity of the ventilatory control system to fluctuations in blood gases. Individuals with a high loop gain exhibit an exaggerated ventilatory response to relatively small changes in oxygen (PaO₂), and carbon dioxide (PaCO₂) levels, increasing the risk of recurrent apneas and unstable breathing pattern during sleep (19, 39). The arousal threshold further modulates this response by determining the level of inspiratory effort required to induce awakening during upper airway obstruction. Both excessively low and excessively high arousal thresholds may worsen disease expression, either by promoting frequent respiratory instability through recurrent awakenings or by prolonging obstructive events and increasing the risk of sustained hypoxemia (16, 40).

These physiological mechanisms are particularly relevant in patients with NCFB, who frequently experience chronic gas exchange abnormalities. Sustained hypoxia and hypercapnia may increase respiratory drive and lower the threshold for arousal, making patients more susceptible to sleep disruption even in response to relatively mild airway obstruction (41). Moreover, recurrent nocturnal hypoxemia further impairs protective ventilatory responses and contributes to progressive instability of respiratory control during sleep (42).

Alterations in sleep architecture commonly observed in BE may further reinforce this process. Patients with BE typically exhibit reduced total sleep time, lower sleep efficiency, increased time spent in light sleep (NREM stage 1), and reduced REM sleep (18, 19). These abnormalities not only reflect the consequences of nocturnal respiratory disturbances but may also exacerbate ventilatory instability by promoting recurrent arousals and preventing the restorative effects of consolidated sleep. Consequently, impaired respiratory control, sleep fragmentation, and abnormal sleep architecture may interact in a self-reinforcing cycle that increases the susceptibility of patients with BE to SDB while potentially worsening the clinical manifestations of both syndromes.

#### 2.2.3 Nocturnal symptoms

Nocturnal symptoms represent an important clinical interface between BE and SDB, since they directly contribute to sleep disruption while further aggravating respiratory instability. Chronic productive cough, a hallmark of BE, is one of the main reasons of disturbed sleep. Recurrent nocturnal coughing episodes induce abrupt arousals and fluctuations in ventilation and PaCO₂, which are particularly detrimental in individuals with a high loop gain, where even minor changes in respiratory drive can perpetuate ventilatory instability (37, 43). Consequently, nocturnal cough not only disrupts sleep continuity but may also facilitate the occurrence and persistence of sleep-disordered breathing in susceptible individuals (16, 38).

The clinical relevance of these mechanisms is supported by studies showing that increased cough frequency and sputum production are consistently associated with worse sleep quality, sleep disturbances, daytime sleepiness and greater nocturnal symptom burden in patients with BE (19, 44, 45). Therefore, nocturnal cough should be considered not merely a symptom of BE but also a potential contributor to the bidirectional interaction between BE and SDB.

Additional comorbid conditions may further increase susceptibility to SDB. Obesity promotes upper airway narrowing through fat deposition within pharyngeal structures and increased upper airway edema, thereby facilitating airway collapse during sleep (34). Gastroesophageal reflux disease (GERD), another common comorbidity in BE, may further exacerbate this interaction, since reflux-induced upper airway inflammation contributes to pharyngeal narrowing, while the marked negative intrathoracic pressure generated during obstructive respiratory events favours reflux episodes, establishing a self-perpetuating bidirectional relationship between GERD and SDB (17, 46, 47). Moreover, GERD may induce bronchoconstriction and trigger cough, thereby worsening nocturnal respiratory symptoms and further compromising airway stability (17, 48).

Taken together, nocturnal cough, mucus retention, obesity, and GERD represent interconnected clinical factors that not only impair sleep quality but may also amplify upper airway dysfunction and ventilatory instability, reinforcing the reciprocal relationship between bronchiectasis and sleep-disordered breathing.

## 3 Clinical consequences

Beyond respiratory dysfunction, SDB may contribute to clinically relevant neurophysiological and cognitive consequences in patients with BE through the interaction of sleep fragmentation, intermittent hypoxemia, impaired gas exchange, and systemic inflammation. Rather than acting independently, these mechanisms may perpetuate one another, linking nocturnal respiratory disturbances with impaired daytime functioning and reduced quality of life.

Recurrent nocturnal hypoxemia and respiratory instability promote repetitive arousals and fragmentation of sleep architecture, particularly reducing restorative deep sleep and rapid eye movement (REM) sleep, which are essential for cognitive processing and emotional regulation (49, 50). In parallel, sustained sympathetic activation, oxidative stress, and chronic systemic inflammation, features shared by both BE and SDB, may further impair central nervous system function through persistent neuroinflammatory signalling (51, 52). Moreover, circulating pro-inflammatory cytokines have been associated with neurocognitive dysfunction, mood disturbances, impaired daytime performance, and reduced cognitive efficiency, providing a biological link between chronic respiratory disease and neurological outcomes (53, 54).

Hypercapnia and chronic impairment of gas exchange may further compromise cerebral physiology, contributing to symptoms such as morning headaches, fatigue, impaired attention, and reduced alertness. Evidence from SDB, specifically obstructive sleep apnea (OSA), demonstrates that intermittent hypoxia and sleep fragmentation are associated with deficits in executive function, processing speed, episodic memory, and attention, while greater depressive symptom burden is associated with poorer cognitive performance (14). Although these findings cannot be directly extrapolated to BE, they provide a plausible mechanistic framework through which coexisting SDB may contribute to neurocognitive dysfunction in this population.

Evidence specifically evaluating cognitive function in bronchiectasis remains limited but suggests that neuropsychological impairment may represent a clinically relevant manifestation of the disease. Patients with BE have demonstrated lower verbal and performance test scores than healthy controls, with poorer cognitive performance being associated with lower oxygen saturation and greater depressive symptoms (55). Moreover, sleep disturbances and depressive symptoms are highly prevalent in BE, particularly among patients with more advanced disease, supporting the concept that chronic sleep disruption may contribute to both neurophysiological and psychological impairment (56). These observations suggest that cognitive dysfunction in BE is likely multifactorial, reflecting the combined effects of chronic respiratory impairment, nocturnal hypoxemia, sleep disruption, systemic inflammation, and mood disorders rather than structural lung disease alone.

Indeed, sleep quality does not consistently correlate with disease severity or the extent of structural lung damage, indicating that additional factors, including age, chronic cough, sputum production, comorbidities, and coexisting SDB, substantially influence sleep impairment in patients with BE (19, 20). Consistent with this observation, no significant association has been demonstrated between sleep quality and radiological disease severity (19, 20).

Among these potentially modifiable factors, SDB, and specifically OSA, appears to play a particularly important role by contributing substantially to nocturnal hypoxemia in patients with BE. Measures of oxygen desaturation, including peripheral oxygen saturation (SpO₂) nadir, oxygen desaturation index, and the percentage of sleep time spent with SpO₂ < 90%, correlate more closely with obstructive respiratory events than with the severity of the underlying BE. This observation is clinically relevant because nocturnal hypoxemia has been associated with increased mortality in BE (57).

Overall, the available evidence supports the concept that SDB should be considered a potentially treatable trait in BE, not only because of its respiratory consequences but also because of its possible contribution to neurocognitive dysfunction, daytime symptoms, and adverse clinical outcomes. Integrating the underlying pathophysiological mechanisms with clinical assessment and rehabilitation strategies may therefore improve the comprehensive management of patients with bronchiectasis. Table 1 summarizes these relationships and their potential implications for clinical practice.

**Table 1:** Clinical consequences of SDB in BE and implications for rehabilitation practice.

Pathophysiological domain	Key mechanisms	Clinical consequences	Rehabilitation implications	Practical recommendations
Chronic inflammation and infection	Airway inflammation, microbiome dysbiosis, recurrent infection, intermittent hypoxia	More frequent BE exacerbations, BE progression, systemic inflammation	Reduced exercise tolerance, higher symptom burden	Airway clearance techniques; Anti-inflammatory strategies; Monitor exacerbation frequency; consider multidisciplinary management
Nocturnal hypoxemia	Intermittent and sustained oxygen desaturation, impaired gas exchange	Fatigue, morning headaches, increased mortality risk	Reduced participation in rehabilitation, impaired recovery	Screen for nocturnal hypoxemia; monitor SpO₂ during sleep and exercise; evaluate need for supplemental oxygen/ PAP/NIV
Ventilatory instability (high loop gain)	Exaggerated ventilatory response, recurrent arousals, unstable breathing control	Sleep fragmentation, recurrent apneas/hypopneas	Poor sleep quality affecting rehabilitation adherence and outcomes	Cardio-pulmonary study during sleep; tailor PAP/NIV therapy
Sleep fragmentation and altered sleep architecture	Recurrent arousals, reduced REM and deep sleep	Daytime sleepiness, impaired cognition, reduced quality of life	Reduced engagement in rehabilitation, impaired learning and self-management	Include sleep quality assessment; patient education on sleep hygiene; integrate behavioral strategies
Nocturnal symptoms (cough, sputum)	Nocturnal cough, mucus retention, GERD-related mechanisms	Sleep disruption, daytime fatigue, symptom worsening	Reduced participation in rehabilitation sessions	Schedule airway clearance before sleep; optimize secretion management; address GERD
Neurocognitive and psychological effects	Hypoxia, inflammation, autonomic dysregulation	Impaired attention, memory deficits, mood disorders, reduced self-management	Reduced adherence to rehabilitation and treatment	Screen for cognitive and psychological impairment; integrate psychological support; simplify treatment plans
Comorbidities (OSA, COPD, obesity, GERD)	Overlapping pathophysiological pathways, airway collapsibility	Increased disease complexity, worse prognosis	Greater rehabilitation complexity, need for individualized care	Systematic screening for SDB; personalized rehabilitation programs; multidisciplinary care pathways
Under-recognition of SDB	Atypical symptoms, poor correlation with classical OSA features	Delayed diagnosis, untreated comorbidity	Suboptimal rehabilitation outcomes	Implement routine screening beyond symptoms; consider objective sleep studies in high-risk patients

## 4 Therapeutic approaches

Currently, evidence guiding the management of BE associated with SDB remains limited, and most available data derive from studies conducted in patients with cystic fibrosis (CF). Consequently, although these findings provide important pathophysiological and therapeutic insights, their applicability to NCFB should be interpreted with caution.

Nocturnal hypercapnia commonly manifests with dyspnea, morning headaches, and sleep fragmentation, all of which may further impair daytime functioning and exercise tolerance. A randomized placebo-controlled trial demonstrated that a 6-week course of non-invasive ventilation (NIV) improved nocturnal PaCO₂, quality of life as well as exercise capacity (58). Additional studies reported reductions in morning headaches together with subjective improvements in sleep quality during long-term NIV treatment (59). These findings suggest that nocturnal ventilatory support may alleviate symptoms that negatively influence daily functioning and may therefore represent a relevant component of comprehensive disease management.

The physiological *rationale* for NIV is supported by studies in advanced CF, where nocturnal ventilatory assistance reduces the work of breathing by approximately 60%–80% (60, 61). NIV has been shown to decrease inspiratory esophageal pressure swings and the diaphragmatic pressure–time product, two surrogate markers of respiratory muscle effort (62). Furthermore, subjective comfort during pressure support ventilation correlates closely with objective measures of respiratory muscle unloading, supporting the clinical relevance of these physiological improvements (62). Overall, NIV is associated with reduced tachypnea, improved oxygenation, dyspnea relief and generally good treatment compliance (63). However, the available evidence is not entirely consistent. While symptomatic improvements are reproducibly reported, several studies have failed to demonstrate sustained reductions in PaCO₂ (63, 64). This suggests that the clinical benefits of NIV may extend beyond correction of hypercapnia alone. Nevertheless, by increasing tidal volume and minute ventilation during sleep, NIV consistently improves nocturnal gas exchange and patient comfort (62, 65).

Long-term observational studies further support the potential role of NIV in chronic suppurative lung disease. Wadsworth et al. reported improvements in lung function together with attenuation of hypercapnia in adults with CF receiving nocturnal NIV, with approximately 50% survival at five years after treatment initiation (66). Similarly, retrospective studies suggest that long-term NIV may contribute to stabilization of lung function over time (67, 68). Although encouraging, these findings derive predominantly from CF cohorts and cannot be directly extrapolated to patients with NCFB.

Evidence specifically evaluating treatment of patients with coexisting BE and OSA remains limited. In a retrospective cohort study, Baskaran et al. evaluated adults with BE and OSA treated with positive airway pressure (PAP) therapy at the Mayo Clinic Center for Sleep Medicine between 2000 and 2020, comparing them with matched OSA controls without BE (7). PAP adherence was comparable between groups, suggesting that BE does not adversely affect long-term treatment compliance. Importantly, patients adherent to PAP therapy showed favourable trends in Bronchiectasis Severity Index (BSI) and Medical Research Council (MRC) dyspnea scores, whereas exacerbations occurred more frequently among patients who were non-compliant to treatment (7). Although these observations require confirmation in prospective studies, they support the hypothesis that effective treatment of SDB may positively influence clinically relevant outcomes in BE.

From a rehabilitation perspective, untreated SDB may represent an important modifiable factor which negatively impacts on functional recovery by contributing to persistent nocturnal hypoxemia, sleep fragmentation, daytime fatigue, reduced exercise tolerance and impaired symptom control. Identification and treatment of SDB may therefore optimize participation in pulmonary rehabilitation and improve patient-reported outcomes, although high-quality evidence supporting specific rehabilitation strategies remains unavailable.

Overall, current evidence suggests that PAP and NIV may provide meaningful physiological and symptomatic benefits in selected patients with BE and SDB. However, available literature remains limited by small sample sizes, retrospective study designs, substantial clinical heterogeneity and the predominance of evidence derived from CF populations. Well-designed prospective studies are needed to determine which patients are most likely to benefit from treatment, to identify predictors of response, and to clarify whether integrating PAP/NIV with pulmonary rehabilitation, sleep optimization, symptom management, psychological support, and self-management interventions can improve long-term patient-centered outcomes.

## 5 Conclusions

The interplay between BE and SDB is complex and likely bidirectional, involving shared pathophysiological mechanisms such as chronic inflammation, oxidative stress, intermittent hypoxia, ventilatory control instability, autonomic dysregulation, and microbiome alterations. These overlapping pathways may contribute not only to disease progression and increased symptom burden, but also to impaired sleep architecture, neurophysiological alterations, and reduced daytime functioning when the two conditions coexist. Current evidence suggests that SDB may represent an underrecognized and potentially modifiable comorbidity in patients with BE. Although available data remain limited, existing studies indicate that interventions such as positive airway pressure (PAP) therapy and non-invasive ventilation (NIV) may improve gas exchange, nocturnal symptoms, and possibly disease-related outcomes (69, 70). However, robust evidence supporting their long-term efficacy in NCFB is still lacking.

Furthermore, emerging evidence suggests that sleep disruption in BE may be associated with clinically relevant psychological, neurophysiological, and cognitive consequences. Although evidence on targeted neuropsychological interventions in bronchiectasis remains scarce, recognizing the potential interaction between sleep disturbances, neurophysiological alterations, mood symptoms, and cognitive functioning may help promote more comprehensive and multidisciplinary approaches to patient care (14). From a clinical perspective, increased awareness and systematic evaluation of sleep disturbances in patients with BE may be warranted, particularly in those with persistent symptoms, nocturnal hypoxemia, frequent exacerbations, or impaired daytime functioning. Early identification and targeted management of SDB could therefore represent an important component of personalized and multidisciplinary BE care.

Future research should focus on well-designed prospective studies to better clarify the prevalence, underlying mechanisms, neurophysiological implications, and optimal management strategies of SDB in BE. A deeper understanding of these interactions may ultimately help identify novel therapeutic targets and improve respiratory, psychological, cognitive, and overall patient outcomes.
